# Group A Streptococcus Induces LAPosomes via SLO/β1 Integrin/NOX2/ROS Pathway in Endothelial Cells That Are Ineffective in Bacterial Killing and Suppress Xenophagy

**DOI:** 10.1128/mBio.02148-19

**Published:** 2019-10-01

**Authors:** Yi-Lin Cheng, Chih-Feng Kuo, Shiou-Ling Lu, Omori Hiroko, Ya-Na Wu, Cheng-Lu Hsieh, Takeshi Noda, Shang-Rung Wu, Robert Anderson, Chiou-Feng Lin, Chia-Ling Chen, Jiunn-Jong Wu, Yee-Shin Lin

**Affiliations:** a Department of Biotechnology and Laboratory Science in Medicine, School of Biomedical Science and Engineering, National Yang Ming University, Taipei, Taiwan; b Institute of Basic Medical Sciences, College of Medicine, National Cheng Kung University, Tainan, Taiwan; c Department of Microbiology and Immunology, College of Medicine, National Cheng Kung University, Tainan, Taiwan; d School of Medicine, I-Shou University, Kaohsiung, Taiwan; e Center for Frontier Oral Science, Graduate School of Dentistry, Osaka University, Osaka, Japan; f Research Institute for Microbial Diseases, Osaka University, Osaka, Japan; g Institute of Oral Medicine, College of Medicine, National Cheng Kung University, Tainan, Taiwan; h Institute of Biological Chemistry, Academia Sinica, Taipei, Taiwan; i Department of Microbiology & Immunology, Dalhousie University, Halifax, Canada; j Department of Microbiology and Immunology, College of Medicine, Taipei Medical University, Taipei, Taiwan; k Graduate Institute of Medical Sciences, College of Medicine, Taipei Medical University, Taipei, Taiwan; l School of Respiratory Therapy, College of Medicine, Taipei Medical University, Taipei, Taiwan; m Center of Infectious Disease and Signaling Research, National Cheng Kung University, Tainan, Taiwan; College of Veterinary Medicine, Cornell University; Montana State University-Bozeman; Chang Gung University

**Keywords:** group A streptococcus, LC3-associated phagocytosis (LAP), xenophagy, reactive oxygen species (ROS), endothelial cells

## Abstract

Group A streptococcus (GAS) is an important human pathogen which can cause fatal diseases after invasion into the bloodstream. Although antibiotics and immune surveillance are the main defenses against GAS infection, GAS utilizes internalization into cells as a major immune evasion strategy. Our previous findings revealed that light chain 3 (LC3)-associated single membrane GAS-containing vacuoles in endothelial cells are compromised for bacterial clearance due to insufficient acidification after fusion with lysosomes. However, the characteristics and the activation mechanisms of these LC3-positive compartments are still largely unknown. In the present study, we demonstrated that the LC3-positive GAS is surrounded by single membrane and colocalizes with NADPH oxidase 2 (NOX2) complex but without ULK1, which are characteristics of LC3-associated phagocytosis (LAP). Inhibition of NOX2 or reactive oxygen species (ROS) significantly reduces GAS multiplication and enhances autolysosome acidification in endothelial cells through converting LAP to conventional xenophagy, which is revealed by enhancement of ULK1 recruitment, attenuation of p70s6k phosphorylation, and formation of the isolation membrane. We also clarify that the inactivation of mTORC1, which is the initiation signal of autophagy, is inhibited by NOX2- and ROS-activated phosphatidylinositol 3-kinase (PI3K)/AKT and MEK/extracellular signal-regulated kinase (ERK) pathways. In addition, streptolysin O (SLO) of GAS is identified as a crucial inducer of ROS for β1 integrin-mediated LAP induction. After downregulation of β1 integrin, GAS multiplication is reduced, accompanied with LAP inhibition and xenophagy induction. These results demonstrate that GAS infection preferentially induces ineffective LAP to evade xenophagic killing in endothelial cells through the SLO/β1 integrin/NOX2/ROS pathway.

## Abstract (precis)

Our previous reports showed that the LC3-associated GAS-containing single membrane vacuoles are inefficient for bacterial clearance in endothelial cells, which may result in bacteremia. However, the characteristics and the induction mechanisms of these LC3-positive vacuoles are still largely unknown. Here we provide the first evidence that these LC3-positive GAS-containing single membrane compartments appear to be LAPosomes, which are induced by NOX2 and ROS. Through NOX2- and ROS-mediated signaling, GAS preferentially induces LAP and inhibits bacteriostatic xenophagy in endothelial cells. We also provide the first demonstration that β1 integrin acts as the receptor for LAP induction through GAS-produced SLO stimulation in endothelial cells. Our findings reveal the underlying mechanisms of LAP induction and autophagy evasion for GAS multiplication in endothelial cells.

## INTRODUCTION

Group A streptococcus (GAS), also known as Streptococcus pyogenes, is a human-specific pathogen which causes a wide variety of diseases. The disease manifestations range from localized infection, such as pharyngitis, impetigo, and scarlet fever, to life-threatening invasive diseases, including bacteremia, cellulitis, necrotizing fasciitis, and streptococcal toxic shock syndrome and even postinfectious autoimmune disorders (1, 2). Despite the availability of antibiotics, GAS still causes an estimated half million deaths globally every year (1–3). Besides the problem of antibiotic resistance, GAS also contains multitude of surface-bound and secreted virulence factors against human immune defenses (1, 4, 5).

One of the critical mechanisms by which GAS evades immune surveillance and antibiotic killing is internalization into cells, even though GAS is considered an extracellular bacterium (6–8). Nevertheless, host cells also possess a defense mechanism, i.e., xenophagy, which is defined as the category of autophagy specifically directed at invasive microorganisms (9–11). The mechanism of xenophagy during GAS infection is well established in epithelial cells. Following GAS internalization into epithelial cells by endocytosis, the secreted pore-forming toxin, streptolysin O (SLO), damages the endosomal membrane, resulting in GAS escape into the cytoplasm, whereupon GAS in cytosol is effectively recognized by the ubiquitination system. Recruitment of autophagic machinery to ubiquitin-tagged GAS results in formation of the double-membrane autophagosome structure. Subsequent fusion of the GAS-containing autophagosome with lysosomes, in which acidification occurs, leads to enzymatic degradation of GAS in epithelial cells (12–15).

However, in contrast to the case with epithelial cells, our previous studies showed that the xenophagy induced by GAS in endothelial cells, which form the last-ditch barrier against GAS invading into blood vessels, has intrinsic defects (14, 16, 17). GAS-containing light chain 3 (LC3)-decorated vacuoles do not maintain a sufficiently acidic environment for lysosomal enzyme activity after fusion with lysosomes. Consequently, GAS is not degraded but actually multiplies in endothelial cells (17). We found that in spite of the recruitment of LC3, there was no autophagosomal double-membrane formed around GAS but only single-membrane structures in endothelial cells (14). These LC3-positive compartments also showed lower levels of ubiquitin and autophagy-related protein recruitment, including ULK1, Atg14, and Atg9, in endothelial cells than in epithelial cells (14). The mechanism by which GAS infection preferentially induces the LC3-positive single-membrane compartment in endothelial cells is still largely unknown.

The above-described observations suggest that the LC3-positive vacuoles seen in endothelial cells may not be autophagosomes but LC3-associated phagosomes (LAPosomes), which have mainly been described for phagocytes (18–20). Besides phagocytes, LC3-associated phagocytosis (LAP) has also been reported for nonphagocytic cells, such as fibroblasts and epithelial cells which have internalized bacteria and entotic cells, respectively, by endocytosis (21, 22). While LAP shares similarities with canonical autophagy, there are several divergences (18, 19). One significant difference is the mechanism of activation (18). Autophagy is promoted by AMP-activated protein kinase (AMPK) activation or inhibition of mTORC1 followed by phosphorylation of ULK1 (23). In contrast to conventional autophagy, LAP induction is independent of the AMPK-mTORC1-ULK1 axis but relies on activation of ligand-receptor signaling (18, 24–27). Toll-like receptor (TLR), Fc receptor, immunoglobulin, TIM4, Dectin-1, and β2 integrin have been reported to participate in LAP initiation through stimulation by varied ligands such as pathogen moieties, dead cells, and immune complexes (27–32). The other unique intermediate stage for LAP is the generation of reactive oxygen species (ROS) by NADPH oxidase 2 (NOX2) complex assembly that associates with the phagosomal membrane. ROS is required for LC3 lipidation in LAP but is not required for autophagy (18, 19, 24, 25).

In this study, we first characterized the LC3-positive single-membrane compartment which surrounds GAS in endothelial cells by determining the involvement of the LAP-unique factors. Then the underlying mechanism of LAP induction in GAS-infected endothelial cells was also investigated. We found that GAS induces LAP through activation of β1 integrin by its virulence factor SLO, which induces NOX2 recruitment and ROS production, resulting in inhibition of autophagy and enhancement of GAS multiplication in endothelial cells.

## RESULTS

### GAS infection induces LAP formation for GAS multiplication.

**FIG 1:**
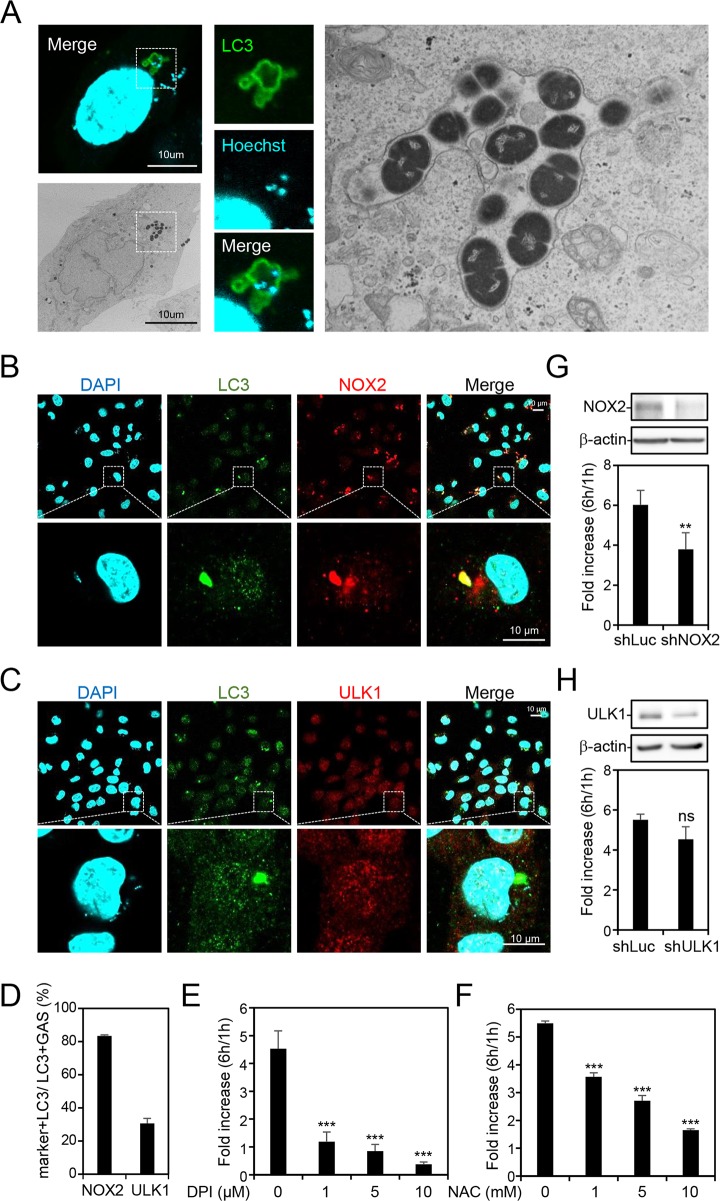
GAS-containing LC3-positive compartments show characteristics of LAP which cause bacterial multiplication in HMEC-1 cells. HMEC-1 cells were infected with GAS at an MOI of 5 for 30 min, and gentamicin was added to kill extracellular bacteria. (A) Cells stably expressing LC3-GFP were fixed and stained with Hoechst for cell nuclear and bacterial DNA staining at 1 h postinfection. Stained specimens were examined by confocal microscopy. After marking the location of cells of interest, the specimen was further fixed and embedded, followed by TEM to analyze the membrane structure of the LC3-positive GAS-containing vacuole in the same cell. (B to D) Cells were collected at 1 h postinfection and stained with anti-NOX2 (B), anti-ULK1 (C), and anti-LC3 antibodies. DAPI was used for cell nuclear and bacterial DNA staining. Images were obtained by confocal microscopy. LC3-positive GAS surrounded with NOX2 or ULK1 (D) was relative to total intracellular LC3-positive GAS. Data represent the means ± SD from three independent experiments, and over 100 cells were counted in each sample. (E and F) Cells were pretreated with diphenyleneiodonium (DPI) (E) and *N*-acetyl-l-cysteine (NAC) (F) for 1 h and infected with GAS at an MOI of 5 for 30 min after removal of the above-named inhibitors and washing with PBS for 2 times. Then gentamicin was added to kill extracellular bacteria in the presence or absence of DPI or NAC. Cells were collected at 1 and 6 h postinfection. The colony forming assay was performed to quantify the amounts of bacteria, and the fold values of GAS replication were calculated by normalizing the GAS count at 6 h with that at 1 h postinfection. Data represent the means ± SD from three independent experiments. ***, *P < *0.001 compared to GAS-infected group without inhibitor. (G and H) NOX2 (G) and ULK1 (H) expression was silenced in HMEC-1 cells by using three lentivirus-based shRNAs. Luciferase shRNA (shLuc) was used as a negative control. The expression of NOX2 and ULK1 was detected by Western blot analysis (upper panel). Cells were further infected with GAS at an MOI of 5 for 30 min, and gentamicin was added to kill extracellular bacteria. The colony forming assay was performed to quantify the numbers of bacteria, and the fold values of GAS replication were calculated by normalizing the GAS count at 6 h with that at 1 h postinfection (lower panel). Data represent the means ± SD from three independent experiments. **, *P < *0.01 compared to shLuc-transfected cells. ns, not significant.

To investigate whether GAS infection induces LAP formation, we first examined the structure of the LC3-positive GAS-containing compartment by correlative light electron microscopy (CLEM) in human microvascular endothelial cell line 1 (HMEC-1) infected with strain NZ131 (M49 serotype) of GAS. The results confirmed that GAS is surrounded by LC3-positive single membrane structures which are a hallmark of LAP (Fig. 1A). LAP is also characterized by unique initiation factors, NOX2 complex and ROS, but is independent of the AMPK-mTORC1-ULK1 axis, which is responsible for conventional autophagy initiation (18). Therefore, we determined the colocalization of NOX2 and ULK1 to LC3-positive GAS by immunostaining and confocal microscopy following infection of GAS for 1 h. The image results showed that the NOX2 puncta were significantly concentrated on LC3-positive GAS (Fig. 1B), whereas ULK1 was mostly diffused in the cytoplasm (Fig. 1C). The quantitative results showed that about 80% of LC3-positive GAS colocalized with NOX2, whereas ULK1 was expressed on only about 30% of LC3-positive GAS (Fig. 1D). We further used the NOX2-selective inhibitor diphenyleneiodonium (DPI) and ROS scavenger *N*-acetyl-l-cysteine (NAC) to examine the effect of LAP on GAS multiplication. After treatment with DPI or NAC, GAS multiplication as detected by colony forming assay was reduced dose dependently in HMEC-1 cells (Fig. 1E and F). Knockdown of NOX2 by lentivirus-based short hairpin RNA (shRNA) also inhibited GAS growth, but there was no significant influence on GAS replication after knockdown of ULK1 (Fig. 1G and H). These results demonstrate that GAS mainly induces LAP in endothelial cells, which may provide a benefit for its multiplication.

### Inhibition of NOX2 and ROS enhances acidification of LC3-decorated GAS-containing structures.

**FIG 2:**
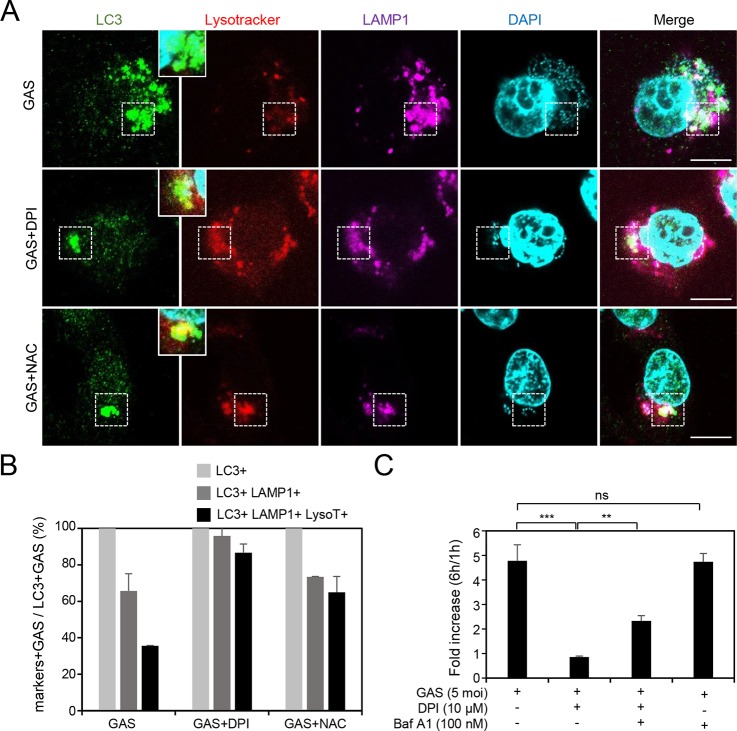
Inhibition of NOX2 and ROS enhances acidification of the lysosome-fused GAS-containing compartment, which is surrounded by LC3. (A and B) After prestaining with LysoTracker (200 nM) in the presence or absence of DPI (10 μM) or NAC (10 mM) for 1 h, the HMEC-1 cells were infected with GAS at an MOI of 5 for 30 min, and then gentamicin was added to kill extracellular bacteria. Cells were fixed at 3 h postinfection and stained with anti-LC3 and anti-LAMP-1 antibodies. DAPI was used for cell nuclear and bacterial DNA staining. Specimens were examined by confocal microscopy (A). LysoTracker (LysoT) stained strongly in the LC3- and LAMP-1-positive compartment after treatment with DPI (10 μM) or NAC (10 mM) in HMEC-1 cells (areas surrounded by a dashed line). Data represent the means ± SD from three independent experiments, and more than 100 cells were counted in each sample (B). Scale bar, 10 μm. (C) HMEC-1 cells with or without 1-h pretreatment with bafilomycin A1 (Baf A1) (100 nM) and/or DPI (10 μM) were infected with GAS at an MOI of 5 for 30 min and then treated with gentamicin to kill extracellular bacteria. Cells were collected at 1 and 6 h postinfection. The colony forming assay was performed to quantify the numbers of bacteria, and the fold values of GAS replication were calculated by normalizing the GAS count at 6 h with that at 1 h postinfection. Data represent the means ± SD from three independent experiments. **, *P < *0.01; ***, *P < *0.001.

Our previous study revealed that the GAS-containing LAPosomes were not acidified sufficiently after fusion with lysosome in endothelial cells, resulting in GAS multiplication (17). Using confocal microcopy in the present study, we found that acidification of the LC3- and LAMP1 (lysosome marker)-positive GAS-containing compartment can be reversed after treatment with DPI and NAC, as indicated by condensed staining of LysoTracker (Fig. 2A). The quantitative results also showed that the percentage of triple positive-GAS was increased after treatment with DPI or NAC (Fig. 2B). Combined treatment with DPI and bafilomycin A1, an inhibitor of autolysosomal acidification, increased the multiplication of GAS compared with that of the DPI-treated group (Fig. 2C). However, bafilomycin A1 alone did not influence GAS multiplication in HMEC-1 cells (Fig. 2C). These results indicate that inhibition of NOX2 and ROS downregulates GAS multiplication by enhancing acidification of LC3-positive GAS-containing vacuoles which fuse with lysosomes.

### Inhibition of NOX2 and ROS enhances the conventional autophagy pathway during GAS infection in HMEC-1 cells.

**FIG 3:**
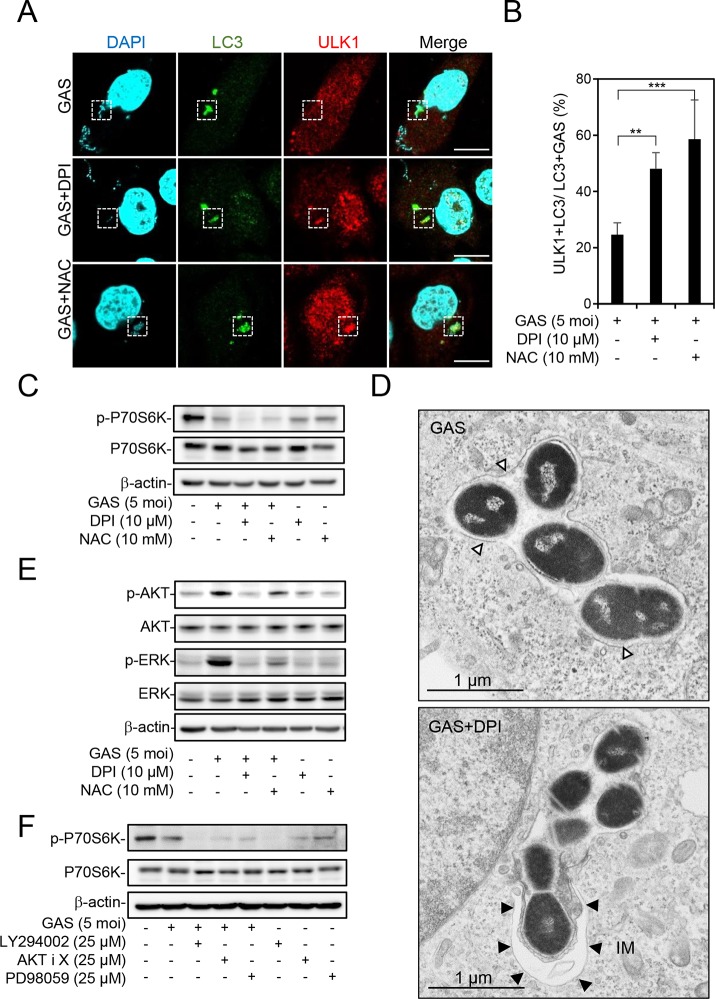
Inhibition of NOX2 and ROS switches LAP to autophagy during GAS infection. (A to E) In the presence or absence of DPI (10 μM) or NAC (10 mM) for 1 h, HMEC-1 cells were infected with GAS at an MOI of 5 for 30 min, and then gentamicin was added to kill extracellular bacteria. (A and B) Cells were fixed at 1 h postinfection and stained with anti-ULK1 and anti-LC3 antibodies. DAPI was used for cell nuclear and bacterial DNA staining. Specimens were observed by confocal microscopy (A). Scale bar, 10 μm. LC3-positive GAS surrounded with ULK1 was relative to total intracellular LC3-positive GAS (B). Data represent the means ± SD from three independent experiments, and over 100 cells were counted in each sample. **, *P < *0.01; ***, *P < *0.001. (C) Cells were collected at 1 h postinfection, followed by Western blot analysis to determine the protein expression levels of phosphorylated p70s6k Thr389 and total p70s6k. (D) Cells were fixed at 1 h postinfection, and conventional TEM was performed to observe the membrane structure of GAS-containing vacuoles. White arrowheads indicate the single-membrane structure; black arrowheads indicate the double-membrane structure. IM, isolation membrane. (E) Cells were collected at 1 h postinfection, followed by Western blot analysis to determine the protein expression levels of phosphorylated AKT Ser473, total AKT, phosphorylated ERK1/2 Thr202/Tyr204, and total ERK. (F) After pretreatment or no pretreatment with LY294002 (25 μM), AKT inhibitor X (25 μM), and PD98059 (25 μM) for 1 h, cells were infected with GAS at an MOI of 5 for 30 min, and then gentamicin was added to kill extracellular bacteria. Cells were collected at 3 h postinfection, followed by Western blot analysis to determine protein expression levels of phosphorylated p70s6k Thr389 and total p70s6k. β-Actin was used as an internal control for Western blot analysis.

We speculated that inhibition of NOX2 and ROS switches the LAP pathway to the autophagy pathway, so that LC3-positive GAS-containing vacuoles, which become autophagosomes, can be acidified after fusion with lysosomes. To investigate this hypothesis, HMEC-1 cells were infected with GAS in the presence or absence of DPI or NAC, followed by the measurement of the recruitment of ULK1, a marker of the autophagic initiation complex, by confocal microscopy. The image results showed that ULK1 formed puncta and colocalized with LC3, which was recruited to GAS after treatment with DPI and NAC (Fig. 3A). The percentage of ULK1 and LC3 doubly positive GAS organisms was also increased by treatment with DPI and NAC (Fig. 3B). Furthermore, treatment with DPI and NAC enhanced the dephosphorylation of p70-s6 kinase (p70s6k) at Thr389, which is a downstream substrate of mTORC1, indicating that mTORC1 was inactivated and resulted in autophagy induction (Fig. 3C). Furthermore, transmission electron microscopy (TEM) images revealed autophagosome double-membrane-engulfed GAS after treatment with DPI, demonstrating inhibition of NOX2 promoted autophagosome formation (Fig. 3D). These data indicate that the NOX2-ROS-induced LAP pathway attenuates signaling for autophagy induction and autophagosome formation. To further clarify the underlying mechanism, we examined the involvement of two main signaling pathways, namely, the phosphatidylinositol 3-kinase (PI3K)/AKT and MEK/extracellular signal-regulated kinase (ERK) pathways, which may activate mTORC1 in LAP-mediated inhibition of autophagy (33, 34). The phosphorylation of AKT at Ser473 and ERK1/2 at Thr202/Tyr204 was detected by Western blotting. The results showed that GAS infection upregulated phosphorylation of AKT and ERK, which was inhibited by DPI and NAC (Fig. 3E). Moreover, the phosphorylation of p70s6k was blocked by pharmacological inhibition of PI3K, AKT, and MEK/ERK by LY294002, AKT inhibitor X, and PD98059, respectively, during GAS infection (Fig. 3F). These results indicate that NOX2 and ROS signaling induces LAP formation and meanwhile attenuates the autophagy pathway by triggering PI3K/AKT- and ERK-mediated mTORC1 activation.

### GAS virulence factor SLO induces LAP formation.

**FIG 4:**
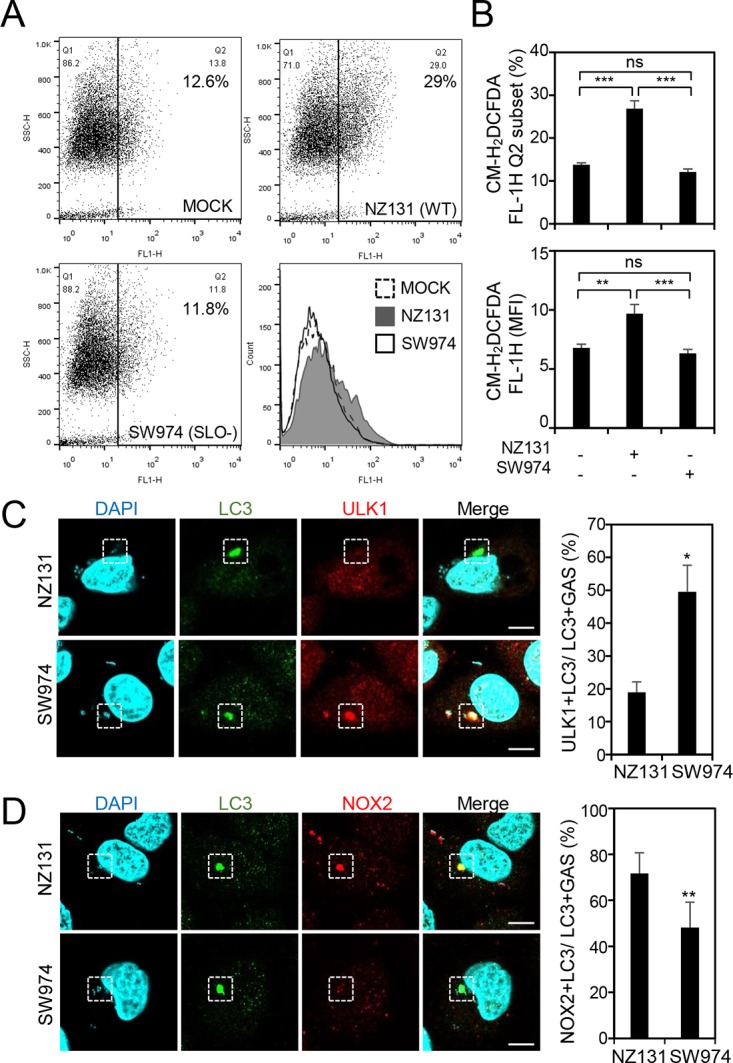
GAS virulence factor SLO is a critical inducer for LAP induction. HMEC-1 cells were infected with wild-type or SLO deletion (SW974) NZ131 at an MOI of 5 for 30 min, and then gentamicin was added to kill extracellular bacteria. (A and B) Cells were collected at 1 h postinfection, followed by staining of CM-H_2_DCFDA and flow cytometric analysis. The data are shown as the dot plots and the histogram (A), as well as quantified by the percentage of the high-fluorescence subset (Q2) and the mean fluorescence intensity (MFI) (B). Data represent the means ± SD from three independent experiments. **, *P < *0.01; ***, *P < *0.001. (C and D) Cells were collected at 1 h postinfection and stained with anti-ULK1 (C), anti-NOX2 (D), and anti-LC3 antibodies. DAPI was used for cell nuclear and bacterial DNA staining. Images were obtained by confocal microscopy. Scale bars, 10 μm. LC3-positive GAS surrounded with ULK1 (C) or NOX2 (D) was relative to total intracellular LC3-positive GAS. Data represent the means ± SD from three independent experiments, and over 100 cells were counted in each sample. *, *P < *0.05, and **, *P < *0.01, compared to wild-type NZ131-infected cells.

A previous study showed that listeriolysin O (LLO), a pore-forming toxin of Listeria monocytogenes, is necessary for LAP formation in macrophages. LLO forms pores on the membrane of LAP to uncouple the pH gradient, resulting in insufficient acidification of LAP followed by bacterial multiplication (35). Hence, we hypothesized that streptolysin O (SLO) of GAS, which is homologous to LLO, is a major factor to induce LAP in endothelial cells. In addition, another study also showed that ROS generation is significantly blocked by a SLO mutant strain of GAS compared to the case with the wild-type strain (36). To verify this hypothesis, ROS generation was determined by carboxymethyl-H_2_-dichlorofluorescein diacetate (CM-H_2_DCFDA) staining followed by flow cytometry analysis after wild-type NZ131 strain and SLO-mutant NZ131 strain (SW974) infection. Compared to ROS production in NZ131-infected cells, ROS production was markedly reduced in SW974-infected cells, as analyzed by either the percentage of ROS-producing cells or the mean fluorescence intensity (Fig. 4A and B). The level of ULK1 recruitment to LC3-positive GAS was higher in SW974-infected cells than in wild-type NZ131-infected cells (Fig. 4C). Conversely, the expression of NOX2 colocalized to LC3-decorated GAS was reduced in SW974-infected cells compared to that in wild-type NZ131-infected cells (Fig. 4D). These results demonstrate that SLO is a crucial virulence factor contributing to formation of LAP, which is insufficiently acidified in endothelial cells during GAS infection.

### β1 integrin acts as a receptor for LAP induction in endothelial cells during GAS infection.

**FIG 5:**
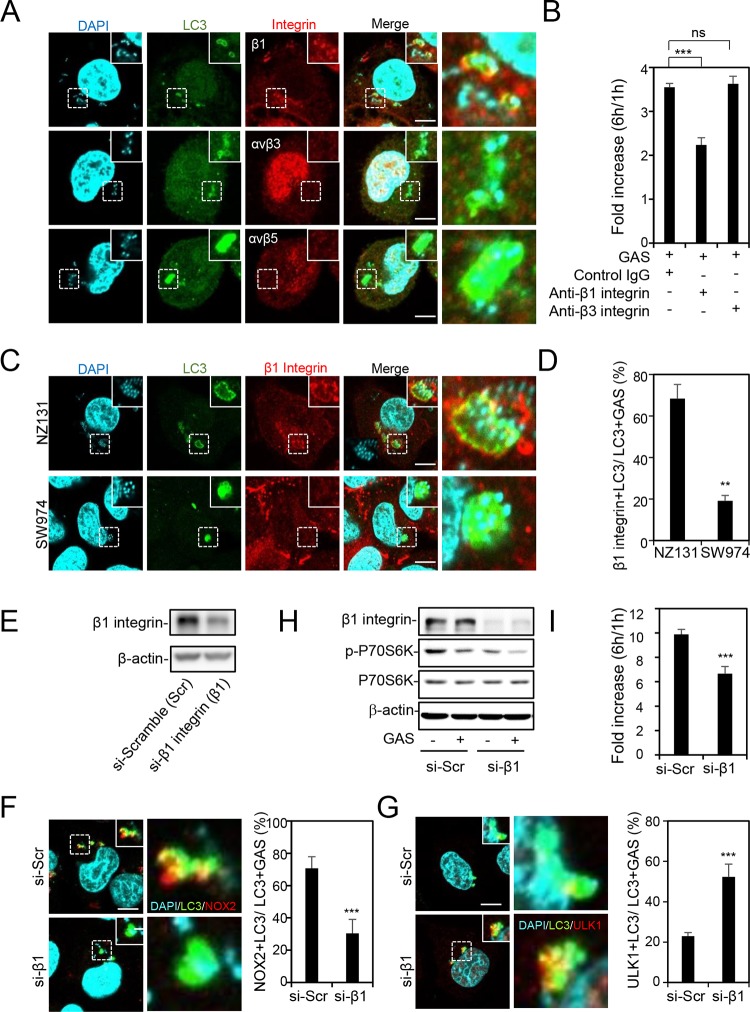
β1 integrin induces LAP through stimulation of SLO. (A) HMEC-1 cells were infected with GAS at an MOI of 5 for 30 min, and gentamicin was added to kill extracellular bacteria. Cells were fixed at 1 h postinfection and stained with anti-β1 integrin, anti-αvβ3 integrin, anti-αvβ5 integrin, and anti-LC3 antibodies. DAPI was used for cell nuclear and bacterial DNA staining. Specimens were examined by confocal microscopy. Scale bars, 10 μm. (B) After pretreatment with mouse control IgG antibody (50 μg/ml), anti-β1 integrin antibody (50 μg/ml), or anti-αvβ3 antibody (50 μg/ml) for 1 h, cells were infected with GAS at an MOI of 5 for 30 min and gentamicin was added to kill extracellular bacteria. The colony forming assay was performed to quantify the numbers of bacteria, and the fold values of GAS replication were calculated by normalizing the GAS count at 6 h with that at 1 h postinfection. Data represent the means ± SD from three independent experiments. ***, *P < *0.001. (C and D) Cells were infected with wild-type or SLO deletion (SW974) NZ131 at an MOI of 5 for 30 min, and then gentamicin was added to kill extracellular bacteria. Cells were fixed at 1 h postinfection and stained with anti-β1 integrin and anti-LC3 antibodies. DAPI was used for cell nuclear and bacterial DNA staining. Images were obtained by confocal microscopy (C). Scale bars, 10 μm. LC3-positive surrounded with β1 integrin was relative to total intracellular LC3-positive GAS. Data represent the means ± SD from three independent experiments, and over 100 cells were counted in each sample. *, *P < *0.05, and **, *P < *0.01, compared to wild-type NZ131-infected cells (D). (E) After knockdown of β1 integrin by siRNA for 48 h, cells were collected to detect the expression of β1 integrin by Western blot analysis. Scramble siRNA was used as a negative control. (F to I) After knockdown of β1 integrin by siRNA for 48 h, cells were infected with GAS at an MOI of 5 for 30 min, and then gentamicin was added to kill extracellular bacteria. Scramble siRNA was used as a negative control. Cells were collected at 1 h postinfection and stained with anti-NOX2 (F), anti-ULK1 (G), and anti-LC3 antibodies. DAPI was used for cell nuclear and bacterial DNA staining. Images were obtained by confocal microscopy. Scale bars, 10 μm. LC3-positive GAS surrounded with NOX2 (F) or ULK1 (G) was relative to total intracellular LC3-positive GAS. Data represent the means ± SD from three independent experiments, and over 100 cells were counted in each sample. ***, *P < *0.001 compared to scramble siRNA-transfected cells. Cells were collected at 1 h postinfection followed by Western blot analysis to determine protein expression levels of β1 integrin, phosphorylated p70s6k Thr389, and total p70s6k. β-Actin was used as an internal control (H). The colony forming assay was performed to quantify the numbers of bacteria, and the fold values of GAS replication were calculated by normalizing the GAS count at 6 h with that at 1 h postinfection. Data represent the means ± SD from three independent experiments. ***, *P < *0.001 compared to scramble siRNA-transfected cells (I).

Based on a previous study, the β2 integrin Mac-1 is the critical receptor for LAP induction through activating acid sphingomyelinase and ROS generation by NOX2 in L. monocytogenes-infected macrophages (32). Endothelial cells also express multiple integrins, which can be mainly divided into β1, αvβ3, and αvβ5 integrins (37). Using immunostaining and confocal microscopy, we found only β1 integrin, and not αvβ3 integrin and αvβ5 integrin, colocalized with LC3 which was recruited to GAS (Fig. 5A). The multiplication of GAS was also inhibited by anti-β1 integrin antibody but not anti-β3 integrin antibody (Fig. 5B). In SLO mutant strain-infected cells, the β1 integrin-positive LC3-decorated GAS was dramatically reduced (Fig. 5C and D), implying that SLO may be the ligand of β1 integrin for LAP induction. To further investigate the role of β1 integrin in LAP induction, knockdown of β1 integrin was performed by transfection with short interfering RNA (siRNA) and examined by Western blotting after 48 h (Fig. 5E). The percentage of NOX2-positive LC3-surrounded GAS was decreased and the recruitment of ULK1 to LC3-positive GAS was increased after knockdown of β1 integrin (Fig. 5F and G). Downregulation of β1 integrin also enhanced the inactivation of mTORC1, as demonstrated by reduction of p70s6k phosphorylation (Fig. 5H). GAS growth in β1 integrin knockdown cells was reduced compared with that in control siRNA-transfected cells (Fig. 5I). Taken together, these data reveal that β1 integrin activates LAP and inhibits autophagy for GAS multiplication through SLO stimulation.

## DISCUSSION

**FIG 6:**
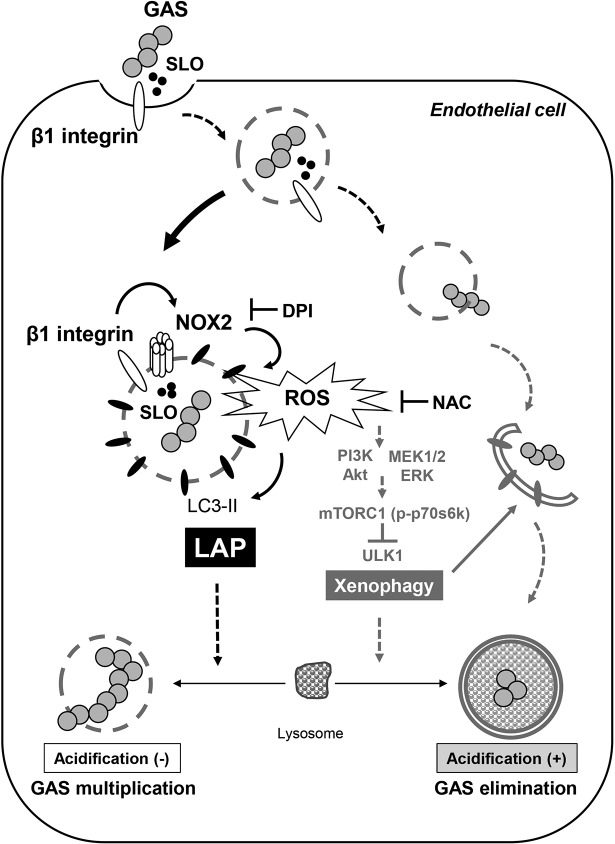
A hypothetical model of the mechanism of LAP induction and autophagy inhibition during GAS infection in endothelial cells. GAS virulence factor SLO activates β1 integrin, followed by NOX2 activation and ROS production for LAP formation, which show insufficient acidification and benefits for GAS multiplication. Simultaneously, NOX2/ROS inhibits autophagy induction, which enhances bacterial clearance and is demonstrated by mTORC1 inactivation and ULK1 recruitment, via activating the PI3K/AKT and MEK1/2/ERK1/2 pathways. These mechanisms provide the strategy for GAS to escape autophagic clearance and to efficiently multiply in endothelial cells.

The results of the present study demonstrate that GAS-secreted SLO activates β1 integrin, followed by NOX2 activation to induce ROS production, which contributes to LAP formation and autophagy inhibition by activating the PI3K/AKT and MEK/ERK pathways to attenuate mTORC1 inactivation. The GAS-containing LAPosome in endothelial cells is not sufficiently acidified after fusion with lysosomes, resulting in loss of function for GAS clearance. The mechanism unveils a novel strategy of GAS to evade host autophagic clearance (Fig. 6).

A cascade of events is required for the compartmentalization of pathogens into LAPosomes and the progression of LAPosome maturation. Numerous pathogens have evolved strategies to promote their survival and proliferation, either by targeting molecules in the LAP pathway by virulence factors or by hiding in the LAPosome to prevent recognition by cytosolic surveillance mechanisms, such as xenophagy. For example, Aspergillus fumigatus and Mycobacterium tuberculosis exclude NOX2 by melanin and bacterial protein CpsA, respectively (38, 39). Leishmania major metalloprotease GP63 cleaves the soluble *N*-ethylmaleimide-sensitive factor attachment protein receptor (SNARE), i.e., VAMP8, which is involved in NOX2 recruitment to phagosomal membranes, diminishing NOX2 complex assembly followed by decreasing LC3 decoration of the membrane (40). Legionella pneumophila inhibits LC3 lipidation by type IV secretion system (T4SS) effector protein RavZ, and Burkholderia pseudomallei prevents fusion of the LAPosome with the lysosome by type III secretion system (T3SS) factors BipD and BopA (41–43). Furthermore, L. monocytogenes and Yersinia pseudotuberculosis subvert LAP and create a replicative niche in the LAPosome to escape xenophagy action (44, 45).

Although GAS induces LAP in endothelial cells, the LAP is ineffective for bacterial elimination. According to a study by Birmingham et al. (35), the pore-forming toxin of L. monocytogenes, LLO, not only induces LAP formation in macrophages but also damages the membrane of LAPosomes. LLO, which creates small pores in the phagosomal membrane to uncouple pH gradients, shows a similar effect on the membrane of the LAPosome (35, 46). Therefore, despite the presence of proton pump v-ATPase on the LAPosomes, the pH of these compartments remains neutral (35). Our current study suggests that SLO of GAS, which is homologous to LLO, may play a similar role in damage of the LAPosomal membrane in endothelial cells. In addition, the process of autophagosome-lysosome fusion is mediated by a variety of proteins, including the small GTPase Rab7, membrane-tethering complexes, and SNAREs (47). Although the mechanism of LAPosome-lysosome fusion has not been fully elucidated, Rab7 has been demonstrated to participate in this process (48). Several studies have demonstrated that intracellular bacteria can manipulate Rab GTPase protein by different virulence factors, including factors in T3SS, T4SS, glyceraldehyde-3-phosphate dehydrogenase (GAPDH), and proteases. These studies suggest the possibility of the involvement of GAS virulence factors, such as cysteine protease SpeB, serine protease SpyCEP, HtrA, and GAPDH, in subversion of Rab7, although these hypotheses need further investigation (49–52).

ROS are generally considered to be critical factors for antimicrobial action (53). Nevertheless, GAS possesses multiple mechanisms to tolerate high ROS levels in the host, including expression of surface-associated ROS resistant factors, intracellular and secreted enzymes for ROS detoxification and ROS-damaged protein repair, metal transporters involved in homeostasis and ROS resistance, and transcription factors responsible for antioxidant gene induction (54). Therefore, while GAS induces ROS production in endothelial cells, GAS may still survive and grow efficiently, and the multiplication of GAS is not increased after treatment with NOX2 and ROS inhibitors DPI and NAC. In contrast, the growth of GAS was dramatically inhibited after treatment with DPI and NAC, resulting from downregulation of the inhibitory signals of autophagy, PI3K-AKT and MEK-ERK. However, we also noticed that GAS alone can still trigger mTORC1 inactivation, indicating that in addition to LAP, GAS infection induces autophagy simultaneously (Fig. 3C). The level of mTORC1 inactivation, which is inhibited by NOX2- and ROS-mediating signaling, may not be sufficient to form autophagosomes, and the existence of galectin-3 around GAS-containing vacuoles blocks ubiquitination, leading to low-level autophagy-related protein recruitment (16). Although the precise mechanism of autophagy initiation during GAS infection in endothelial cells is not fully understood, a recent study revealed that the extracellular bacterium-derived outer membrane vesicles activate AMPK to inhibit mTORC1 for xenophagy activation (55). Whether GAS components play a similar role in xenophagy induction needs further investigation.

Although the antioxidant mechanisms of GAS are relatively well clarified, the mechanisms of ROS induction by GAS in host cells are still unclear. A recent study (36) showed that ROS production was almost totally blocked in SLO- or NAD^+^ glycohydrolase (NADase) mutant GAS infection, but the strain lacking NADase activity still induced ROS generation in cells. These results imply that SLO and NADase protein coordinate to induce ROS production. Moreover, our data showed that depletion of SLO not only blocked ROS but also reduced β1 integrin-mediated LAP, suggesting that SLO might be the ligand of β1 integrin to induce NOX2 activation followed by ROS production. We speculate that the ligand of β1 integrin for LAP induction may not be SLO alone but both SLO and NADase, which have been demonstrated to bind together to form a complex (56).

In the present study, we first identified that β1 integrin could be the receptor for LAP induction in endothelial cells during GAS infection. Although we only examined the intracellular location of β1 integrin by confocal microscopy, the attenuation of GAS multiplication by treatment with anti-β1 integrin antibody suggests that β1 integrin recognizes GAS, especially SLO, on the cell surface to induce LAP formation. During SLO mutant strain infection, the presence of β1 integrin on LC3-associated vacuoles significantly diminished, accompanied by increasing recruitment of ULK1 and decreasing expression of NOX2 on LC3-positive GAS. These findings imply that without SLO stimulation, the LC3-associated compartment is mainly the GAS-containing autophasosome but not β1-mediated LAP. However, the interaction between SLO and β1 integrin directly or indirectly, as well as the involvement of acid sphingomyelinase in β1 integrin-induced NOX2 activation, which plays a role in β2 integrin-induced LAP during L. monocytogenes infection (32), still needs further exploration. Furthermore, inhibition of β1 integrin by siRNA or specific antibody did not totally block the multiplication of GAS, suggesting that β1 integrin is not the sole receptor for GAS-induced LAP. Hence, the involvement of other LAP-activating receptors, such as TLRs, which can also be activated by GAS needs further investigation (31, 57).

## MATERIALS AND METHODS

### Cell culture and reagents.

Human microvascular endothelial cell line 1 (HMEC-1), which was obtained from the Centers for Disease Control and Prevention, USA, retains the morphological, phenotypic, and functional characteristics of normal human microvascular endothelial cells (58). HMEC-1 cells were passaged in culture plates using endothelial cell growth medium M200 (Cascade Biologics) supplemented with 10% fetal bovine serum (FBS), 1 μg/ml of hydrocortisone, 10 ng/ml of epidermal growth factor, 3 ng/ml of basic fibroblast growth factor, and 10 μg/ml of heparin. Cells were cultured at 37°C in 5% CO_2_ and detached with 1,000 U/ml of trypsin and 0.5 mM EDTA for passage when cell confluence reached 80%. Diphenylene iodonium (DPI), *N*-acetyl-l-cysteine (NAC), bafilomycin A1, LY-294,002 hydrochloride (LY294002), AKT inhibitor X (AKTiX), and PD98059 were purchased from Sigma-Aldrich.

### Bacteria.

GAS strain NZ131 (type M49, T14 strain) was a gift from D. R. Martin (New Zealand Communicable Disease Center). GAS was cultured on tryptic soy agar containing 5% defibrinated sheep blood at 37°C. For the infection model, a colony of GAS was inoculated in tryptic soy broth containing 0.5% yeast extract (TSBY; Difco Laboratories) for 18 h and was transferred to fresh broth at a 1:50 dilution for 3 h. The refreshed, early-log GAS cells were harvested by centrifugation (3,500 rpm, 10 min, 4°C) and resuspended in phosphate-buffered saline (PBS). The bacterial density was determined by spectrophotometry using the optical density at 600 nm (OD_600_), and the sample was diluted with PBS to 1 × 10^8^ CFU/ml. Serial dilutions of the samples were plated on TSBY agar, and viable colonies were counted after incubation overnight at 37°C.

### *slo* mutant construction.

The 3,003-bp fragment containing the flanking region of *slo* was amplified by primers SLO-F (5′-GGCGGATCCCAAGATAGAATGCAAG-3′) and SLO-R (5′-GGCGTCGACGGTGCGGTTTAAGATG-3′). To construct the knockout mutant, the 1,302-bp fragment without the open reading frame of *slo* was constructed by overlap PCR and cloned into streptococcal suicide vector pSF152 by restriction endonucleases EcoRI and SalI. Subsequently, the recombinant plasmid was ligated with a chloramphenicol resistance cassette and then transformed into strain NZ131 to generate *slo* knockout strain SW975 by homologous recombination. Southern blot hybridization was used to confirm the gene replacement.

### Infection model.

HMEC-1 cells were plated at 7 × 10^4^ in 24-well plates or at 3 × 10^5^ in 6-well plates and incubated overnight at 37°C. The prepared bacteria were directly added into wells at multiplicities of infection (MOI) of 5, followed by centrifugation at 500 × *g* and 4°C for 5 min to ensure simultaneous infection of cells. After 30 min of incubation at 37°C, the original medium containing bacteria was removed and the cells were washed twice with PBS. Fresh medium containing 125 μg/ml of gentamicin was added to kill extracellular bacteria. The cells were washed twice with PBS and collected at various time points.

### Immunofluorescence staining and confocal microscopy.

Cells were seeded at 7 × 10^4^ in 24-well plates with coverslips overnight at 37°C and infected with GAS as described above. For acid indicator staining, LysoTracker (red DND-99; Invitrogen) was added at a final concentration of 200 nM in medium for 1 h of incubation at 37°C in the dark before GAS infection. At various time points postinfection, the cells were fixed with 4% paraformaldehyde (Santa Cruz) for 20 min at room temperature (RT), permeabilized with 0.1% Triton X-100 (Sigma-Aldrich) for 15 min at RT, blocked with 1% bovine serum albumin (BSA; Sigma-Aldrich) for 15 min at RT, and stained with anti-LC3 (pM036; M152-3; MBL), anti-NOX2 (ab80508; Abcam), anti-ULK1 (cs8054; Cell Signaling Technology), anti-LAMP1 (cs9091; Cell Signaling Technology), anti-β1 integrin (HUTS-4; Millipore), anti-αvβ3 integrin (LM609; Millipore), and anti-αvβ5 (P1F6; Millipore) antibodies at 4°C overnight. After the cells were washed three times with PBS, they were stained with Alexa Fluor-conjugated secondary antibodies (Invitrogen) and 4′,6-diamidino-2-phenylindole (DAPI; (Calbiochem) for nuclear staining for 1 h, and the samples were then analyzed by confocal microscopy (LSM880; Zeiss).

### Colony forming assay for intracellular GAS multiplication.

HMEC-1 cells were seeded at 7 × 10^4^/well in 24-well plates overnight. Cells were pretreated with inhibitors or anti-β1 integrin antibody (6S6; Millipore), anti-β3 integrin antibody (B3A; Millipore), and mouse control IgG1 antibody (MG1-45; BioLegend) for 1 h and infected with GAS as described above. Gentamicin (125 μg/ml) was added to kill extracellular bacteria. At 1 h and 6 h postinfection, cells were washed twice with PBS and lysed with 1 ml/well of sterile H_2_O. After 10 min, 1 ml of GAS-containing sterile H_2_O was transferred to a new microcentrifuge tube, followed by serial dilutions in 0.85% NaCl. The diluted samples were plated on TSBY agar plates. Colonies were counted after 48 h of culture at 37°C. The bacterial count at 1 h postinfection was interpreted as the number of internalized GAS. The fold increase of bacterial number was calculated by normalizing the bacterial number at 6 h postinfection against the internalized bacterial number.

### Western blotting.

HMEC-1 cells were seeded at 3 × 10^5^/well in 6-well plates overnight and infected with GAS as described above. Harvested cells were lysed in lysis buffer containing protease inhibitor cocktail and phosphatase inhibitor cocktail (Sigma-Aldrich). After centrifugation, the lysates were boiled in sample buffer for 10 min. Samples were then subjected to SDS-PAGE and proteins transferred to polyvinylidene difluoride (PVDF) membranes (Millipore). After blocking with 5% skim milk in PBS, the membranes were incubated overnight at 4°C with primary antibodies, including anti-p70s6k antibody (cs9202), anti-phospho-p70s6k antibody (Thr389) (cs9205), anti-AKT antibody (cs9272), anti-phospho-AKT antibody (Ser473) (cs9271), anti-ERK (p44/42 MAPK) antibody (cs9102), anti-phospho-ERK antibody (Thr202/Tyr204) (cs9101), anti-β1 integrin antibody (cs4706), and anti-β-actin antibody (AC-74; Sigma-Aldrich). The above-named antibodies were purchased from Cell Signaling Technology, except for anti-β-actin antibody. After incubation with horseradish peroxidase (HRP)-conjugated secondary antibody (Cell signaling Technology) at RT for 1 h, membranes were soaked in ECL solution (PerkinElmer Life and Analytical Sciences, Inc.) and the images were captured by a luminescence imaging system (LAS-4000; Fujifilm).

### Intracellular ROS detection.

Cells were seeded at 7 × 10^4^/well in 24-well plates overnight. After GAS infection as described above, cells were collected and then coincubated with a cellular reactive oxygen species detection dye, carboxymethyl-H_2_-dichlorofluorescein diacetate (CM-H_2_DCFDA) (C6827; Invitrogen), at a concentration of 5 μM for 5 min at 37°C in the dark. After washing, cells were collected and analyzed using flow cytometry (FACSCalibur; BD Biosciences) with excitation set at 488 nm. The emission was detected with the FL-1 channel, followed by analysis with CellQuest Pro 4.0.2 software (BD Biosciences).

### Transfection with siRNA.

Cells for siRNA transfection were prepared at 4 × 10^5^/well in 6-well plates and incubated overnight. A 10 nM concentration of Scramble (5′-ACTACCGTTGTTATAGGTG-3′) or β1 integrin siRNA (HSS105560; Sigma-Aldrich) was mixed with Lipofectamine RNAiMAX reagent (Thermo Fisher Scientific) in Opti-MEM medium (Gibco) for 5 min at RT and added to the cell culture medium. After 24 h of incubation, cells were trypsinized, reseeded at 3 × 10^5^/well in 6-well plates, and cultured overnight. The siRNA-transfected cells were infected with GAS as described above or collected for Western blot assay to confirm efficient knockdown of β1 integrin.

### Correlative light electron microscopy.

HMEC-1 cells stably expressing LC3-green fluorescent protein (GFP) (14) were cultured on glass-bottom dishes with a grid pattern (P35G-2-14-C-GRID; MatTek) and infected with GAS as described above. At 1 h postinfection, the cells were fixed with 4% formaldehyde in HEPES (pH 7.4) buffer, containing 30 mM HEPES, 100 mM NaCl, and 2 mM CaCl_2_, as well as 1 μg/ml of Hoechst, for 30 min at RT, washed with HEPES buffer, and analyzed by confocal microscopy (FV1000; Olympus). After marking of the cells of interest by correlating with the grid, the same specimens were further fixed with 2% formaldehyde and 2.5% glutaraldehyde in HEPES buffer at 4°C overnight. After washing three times, the samples were incubated with 1% osmium tetroxide and 0.5% potassium ferrocyanide in HEPES buffer for 1 h. The samples were then washed in distilled water, dehydrated in ethanol, and embedded in Epon 812 (TAAB Laboratories Equipment). The embedded cells were cut into ultrathin sections (70 nm thick) and stained with saturated uranyl acetate and Reynolds lead citrate solution. Transmission electron microscopy (TEM) was carried out on a JEOL JEM-1011 instrument equipped with charge-coupled-device (CCD) camera system to acquire micrographs.

### Conventional TEM.

Cells were infected with GAS for 1 h and gently collected by trypsinization and centrifugation and resuspended in phenol red-free medium containing 5% FBS and 40% dextran T2000. A high-pressure freezer (Leica EM HPM100) was used to perform high-pressure fixation of samples, which were kept on ice before fixation. The specimens were then subjected to freeze-substitution at low temperature and embedded in plastic (Epon 812; TAAB Laboratories Equipment). The embedded specimens were cut into ultrathin sections (70 nm thick), followed by staining with saturated uranyl acetate and Reynolds lead citrate solution. Transmission electron microscopy was carried out on a JEOL JEM-1011 to acquire micrographs.

### Statistical analysis.

Data obtained from independent experiments are presented as the means ± standard deviations (SD). Comparisons of two sets of the data were analyzed by an unpaired Student *t* test. For three or more sets of data, statistics were analyzed by one-way analysis of variance (ANOVA) with Tukey’s multiple-comparison posttest. Statistical analysis was performed using Prism version 5 (GraphPad Software). Statistical significance was set at a *P* value of *<*0.05.
