# Erratum for Cheng et al., “Group A *Streptococcus* Induces LAPosomes via SLO/β1 Integrin/NOX2/ROS Pathway in Endothelial Cells That Are Ineffective in Bacterial Killing and Suppress Xenophagy”

**DOI:** 10.1128/mBio.02250-21

**Published:** 2021-08-31

**Authors:** Yi-Lin Cheng, Chih-Feng Kuo, Shiou-Ling Lu, Hiroko Omori, Ya-Na Wu, Cheng-Lu Hsieh, Takeshi Noda, Shang-Rung Wu, Robert Anderson, Chiou-Feng Lin, Chia-Ling Chen, Jiunn-Jong Wu, Yee-Shin Lin

**Affiliations:** a Department of Biotechnology and Laboratory Science in Medicine, School of Biomedical Science and Engineering, National Yang Ming University, Taipei, Taiwan; b Institute of Basic Medical Sciences, College of Medicine, National Cheng Kung University, Tainan, Taiwan; c Department of Microbiology and Immunology, College of Medicine, National Cheng Kung University, Tainan, Taiwan; d School of Medicine, I-Shou University, Kaohsiung, Taiwan; e Center for Frontier Oral Science, Graduate School of Dentistry, Osaka University, Osaka, Japan; f Research Institute for Microbial Diseases, Osaka University, Osaka, Japan; g Institute of Oral Medicine, College of Medicine, National Cheng Kung University, Tainan, Taiwan; h Institute of Biological Chemistry, Academia Sinica, Taipei, Taiwan; i Department of Microbiology & Immunology, Dalhousie University, Halifax, Canada; j Department of Microbiology and Immunology, College of Medicine, Taipei Medical University, Taipei, Taiwan; k Graduate Institute of Medical Sciences, College of Medicine, Taipei Medical University, Taipei, Taiwan; l School of Respiratory Therapy, College of Medicine, Taipei Medical University, Taipei, Taiwan; m Center of Infectious Disease and Signaling Research, National Cheng Kung University, Tainan, Taiwan

## ERRATUM

Volume 10, no. 5, e02148-19, 2019, https://doi.org/10.1128/mBio.02148-19. Hiroko Omori’s first name and surname were placed incorrectly in the original article. The byline should appear as shown above. The original article has been corrected online.

